# Inactivation of the Wnt/β-catenin signaling pathway underlies inhibitory role of microRNA-129-5p in epithelial–mesenchymal transition and angiogenesis of prostate cancer by targeting ZIC2

**DOI:** 10.1186/s12935-019-0977-9

**Published:** 2019-10-21

**Authors:** Zhenming Jiang, Yuxi Zhang, Xi Chen, Pingeng Wu, Dong Chen

**Affiliations:** 1 Department of Urology, The First Hospital of China Medical University, No. 155, Nanjing North Street, Heping District, Shenyang, 110001 Liaoning People’s Republic of China; 2 Department of Urology, People’s Hospital of Datong Hui and Tu Autonomous County, No. 1, Wenhua Road, Qiaotou Town, Datong Hui and Tu Autonomous County, Xining, 810100 Qinghai People’s Republic of China; 3 Department of Pharmacy, The First Hospital of China Medical University, Shenyang, 110001 People’s Republic of China; 4 Central Lab, The First Hospital of China Medical University, Shenyang, 110001 People’s Republic of China

**Keywords:** Prostate cancer, microRNA-129-5p, Zinc-finger protein of the cerebellum 2, Wnt/β-catenin signaling pathway, Epithelial–mesenchymal transition, Angiogenesis

## Abstract

**Background:**

Prostate cancer (PCa) is a common disease that often occurs among older men and a frequent cause of malignancy associated death in this group. microRNA (miR)-129-5p has been identified as an essential regulator with a significant role in the prognosis of PC. Therefore, this study aimed to investigate roles of miR-129-5p in PCa.

**Methods:**

Microarray analysis was conducted to identify PCa-related genes. The expression of miR-129-5p and ZIC2 in PCa tissues was investigated. To understand the role of miR-129-5p and ZIC2 in PCa, DU145 cells were transfected with mimic or inhibitor of miR-129-5p, or si-ZIC2 and the expression of Wnt, β-catenin, E-cadherin, vimentin, N-cadherin, vascular endothelial growth factor (VEGF), and CD31, as well as the extent of β-catenin phosphorylation was determined. In addition, cell proliferation, migration, invasion, angiogenesis, apoptosis and tumorigenesis were detected.

**Results:**

miR-129-5p was poorly expressed and ZIC2 was highly expressed in PCa tissues. Down-regulation of ZIC2 or overexpression of miR-129-5p reduced the expression of ZIC2, Wnt, β-catenin, N-cadherin, vimentin, and β-catenin phosphorylation but increased the expression of E-cadherin. Importantly, miR-129-5p overexpression significantly reduced cell migration, invasion, angiogenesis and tumorigenesis while increasing cell apoptosis.

**Conclusions:**

The findings of the present study indicated that overexpression of miR-129-5p or silencing of ZIC2 could inhibit epithelial–mesenchymal transition (EMT) and angiogenesis in PCa through blockage of the Wnt/β-catenin signaling pathway.

## Background

Prostate cancer (PCa) is a most frequently occurring malignancy among older men [1, 2]. Prostate tumors are usually indolent, but a considerable number of tumors are highly aggressive and often metastasize to bones and other organs, leading to high morbidity and mortality [3, 4]. In addition, PCa is typically marked by a high recurrence rates, whereby about 40% of local PCa cases recur after initial treatment, and the tumor progresses to hormone refractory/castration resistance stage is basically untreatable [5, 6]. Epithelial–mesenchymal transition (EMT) and its reverse process are essential physiological processes during organogenesis and tissue differentiation of normal embryonic development [7, 8]. The EMT process is also a part of cancer pathogenesis including PCa [9]. Angiogenesis is also an important feature of malignancy, and is particularly relevant in the progression to end-stage PCa [10]. There is a need to unravel the molecular events and players that are involved in these mechanisms.

Past studies have highlighted the regulatory role of microRNAs (miRNAs) in PCa pathogenesis. miRNAs regulate post-transcriptional gene expression and their dysregulation is implicated in the development of cancer [11, 12]. It has been previously reported that up-regulated miR-129-5p could reduce EMT and thus functions as a tumor suppressor [13]. Down-regulation of miR-129 has been demonstrated as a valuable prognostic biomarker of PCa proliferation [14]. Zinc-finger protein of the cerebellum (ZIC) 2, identified as a target gene of miR-129-5p in the present study, is the vertebrate homologues of the Drosophila odd-paired (OPA) gene, including ZIC1, ZIC2, ZIC3, ZIC4 and ZIC5, and has been implicated in multiple diseases including cancer [15, 16]. Another study has proved that the RNA levels of ZIC1, ZIC2, ZIC4 and ZIC5 are all induced in Gleason grade 3 embedded in Gleason score (GS) 4 + 3 = 7 PCa [17]. miRNAs have recently become important regulators of EMT in diversity cancers [18]. miRNAs appear to regulate EMT by modulating posttranscriptional components such as EMT-transcription factors, epithelial and mesenchymal genes, or through regulation of key signaling pathways, which in turn modulate cancer progression and metastasis [7, 18]. For instance, overexpression of miR-129-5p attenuated EMT and proliferation in gastric cancer by downregulating the expression of HMGB1 [19]. The canonical Wnt signaling pathway, extensively conserved in the animal kingdom, is essential for embryonic development and adult tissue homeostasis [20]. Moreover, miR-129-5p has been reported to hamper proliferation and invasion of chondrosarcoma cells by blocking the Wnt/β-catenin signaling pathway [21]. Based on the aforementioned evidences, we hypothesize that miR-129-5p played a significant role in PCa pathogenesis via its regulation of ZIC2-mediated Wnt/β-catenin signaling pathway. Therefore, the current study aimed to examine if miR-129-5p could impact EMT and angiogenesis in PCa by regulating ZIC2-mediated Wnt/β-catenin signaling pathway.

## Materials and methods

### Ethical statement

The study was approved by the Institutional Review Board of the First Hospital of China Medical University. Written informed consents were obtained from all patients or their guardians. All study procedures were conducted in accordance with the Declaration of Helsinki. All animal experiments were conducted under the approval of guidelines for the protection and use of experimental animals issued by the National Institutes of Health (NIH), and strictly complied with the principles of completing the experiments with the minimum number of animals and minimizing pain.

### Microarray analysis

The Gene Expression Omnibus (GEO) database (https://www.ncbi.nlm.nih.gov/geo/) was used to identify PCa-related microarray datasets. The “limma” package in the R language was used to analyze differential expression with |log foldchange| > 2 and *p* < 0.05 as the screening threshold of differentially expressed genes (DEGs). The “pheatmap” package was used to construct a heat map of the DEGs. Next, PCa-related genes were selected using the MalaCards database (http://www.malacards.org/). The STRING database (https://string-db.org/) was used to analyze the correlation between known PCa genes and the DEGs obtained. A gene interaction network was constructed using Cytoscape. The TargetScan database (http://www.targetscan.org/vert_71/), miRDB database (http://mirdb.org/miRDB/index.html), mirDIP database (http://ophid.utoronto.ca/mirDIP/index.jsp#r), miRNApath database (http://lgmb.fmrp.usp.br/mirnapath/tools.php) and starBase database (http://starbase.sysu.edu.cn/) were used to predict the miRNAs that regulated the ZIC2 gene, and then the intersection of the results was obtained. The intersection of the results was searched in the microRNA.org database (http://34.236.212.39/microrna/home.do).

### Study subjects

A total of 60 cases of PCa tissues were collected from the PCa patients who had undergone prostatectomy in the Urology Department in the First Hospital of China Medical University from September 2016 to September 2017, with the corresponding 60 adjacent normal tissues taken as controls (all the samples were verified by pathological examination). All subjects had no missing clinical data. The patients included were aged between 54 and 76 years, old, with 26 patients ≥ 70 years old and 34 patients < 70 years old; 46 patients of prostate transverse diameter > 35 mm and 14 patients of prostate transverse diameter < 35 mm; 43 patients of Gleason score ≤ 7 points and 17 patients of Gleason score > 7 points. And 38 patients in I + II stage, 22 patients in IIIA stage of tumor, node, metastases staging [22]. All the 60 cases of PCa patients were diagnosed as primary tumors. Moreover, all the patients had no previous history of PCa-related chemotherapy or radiotherapy. The adjacent normal tissues were pathologically confirmed to be with no tumor cell infiltration and no obvious inflammatory reaction. The collected samples were fixed with 10% formaldehyde, routinely dehydrated, paraffin-embedded, and cut into 4 μm sections for subsequent experiments.

### Immunohistochemistry

The SP-9001 kit (Beijing noble Ryder Technology Co., Ltd., Beijing, China) was used for Immunohistochemistry. The normal and PCa paraffin tissues were allowed to stand at room temperature for 30 min, fixed by 4 °C acetone for 10 min followed by dewaxing and hydration. Next, samples were soaked with 3% H_2_O_2_ for 5–10 min to inhibit endogenous peroxidase activity and sealed with 5% normal goat serum working solution (C1771, Beijing Applygen Technology Co., Ltd., Beijing, China). After incubation for 10–15 min at 37 °C, the sections were probed with rabbit anti-human antibodies to Wnt3a (ab19925, 1:200, Abcam, Cambridge, UK) and β-catenin (ab16051, 1:100, Abcam, Cambridge, UK) overnight at 4 °C. Next, the sections were allowed to stand at room temperature for 30 min and incubated with the secondary antibody, biotinylated goat anti-rabbit antibody to immune globulin (IgG; 1:1000, ab6721, Abcam, Cambridge, UK) for 1 h at 37 °C. Following that, the sections were reacted with horseradish peroxidase (HRP)-labeled streptavidin (0343-10000U, Imunbio Biotechnology Co., Ltd., Beijing, China) for 1 h at 37 °C and with diaminobenzidine (DAB; ST033, Guangzhou Whiga Science and Technology Co., Ltd., Guangzhou, Guangdong, China) for 3–10 min. Subsequently, the sections were counterstained by hematoxylin (Shanghai Fusheng Industrial Co., Ltd., Shanghai, China) for 1 min, immersed in 1% hydrochloric acid alcohol for 10 s, soaked with tap water, and stained for 10 s with 1% ammonia to obtain a blue color. The sections were then dehydrated with conventional gradient alcohol, cleared by xylene, and sealed with neutral balsam. Phosphate buffer solution (PBS) instead of the primary antibody was used as the blank control. Five high-power fields (200×) were randomly selected from each section and 100 cells were counted in each field. Scores were determined as the proportion of positive cells [23]. The positive cells/total cells > 10% was considered as positive (+) and positive cells ≤ 10% was considered as negative (−). Normally, the β-catenin^+^ cells in PCa tissues were mainly in the cytoplasm and nucleus, and poorly expressed in the membrane. The Wnt^+^ cells were mainly in the cytoplasm. The positively stained cells were expressed as brown or tan. Each experiment was carried out three times.

### Dual-luciferase reporter gene assay

Biological prediction website (https://cm.jefferson.edu/rna22/Interactive/) was applied to conduct the target gene analysis for ZIC2 and miR-129-5p. A dual-luciferase reporter gene assay was used to verify whether ZIC2 was a target gene of miR-129-5p. Based on the predicted binding sequence between the 3′untranslated region (3′UTR) of ZIC2 mRNA and miR-129-5p, the target sequence and the mutant sequence were each designed. The target sequence was chemically synthesized and digested by *Xho*I and *Not*I restriction sites. The synthesized fragment was cloned into the PUC57 vector (HZ0087, Shanghai Huzheng Industrial Co., Ltd., Shanghai, China), and recombinant plasmids were identified by DNA sequence assay once positive clones were identified. Next, the recombinant plasmids were subcloned into the psiCHECK-2 vector (HZ0197, Shanghai Huzhen Industrial Co., Ltd., Shanghai, China), transferred into Escherichia coli DH5α cells and amplified. All the plasmids were extracted in accordance with the instructions of plasmid mini-extracting kit Omega (D1100-50T, Beijing Solarbio Science & technology Co., Ltd., Beijing, China). The cells were seeded in a 6-well plate (2 × 10^5^ cell/well) and transfected once they were adherent to the wall. After 48 h, the effect of miR-129-5p on the luciferase activity of FGF3 3′-UTR in cells was detected using the dual-luciferase reporter assay kit (D0010, Beijing Solarbio Science & technology Co., Ltd., Beijing, China). The fluorescence intensity was measured using a Promega Glomax 20/20 luminometer fluorescence detector (E5311, Shanxi Zhongmei Bio-technology Co., Ltd., Xian, Shaanxi, China). Each experiment was repeated three times.

### Cell culture, grouping and transfection

The DU145 PCa cell line (Cell Bank of Shanghai Institute of Cells, Chinese Academy of Science, Shanghai, China) was cultured in Dulbecco’s modified Eagle medium (DMEM) containing 10% fetal bovine serum (FBS) [24] with a mixture of penicillin–streptomycin solution at the ratio of 1:1 (100 U/mL), in a 5% CO_2_ incubator at 37 °C. Cells were detached with 0.25% trypsin and passaged at a ratio of 1:3. Next, the cells were cultured in 6-well plates at a density of 3 × 10^5^ cells/well. When the cells reached 70–80% confluence, the following experiments were carried out.

The cells at passage three were treated with trypsin, seeded in 24-well plates, and cultured until they grew into monolayers. These cells were then grouped as follows: the blank group (DU145 cells transfected without any sequence), the negative control (NC) group (DU145 cells transfected with scramble siRNA), the miR-129-5p mimic group (DU145 cells transfected with miR-129-5p mimic plasmid), the miR-129-5p inhibitor group (DU145 cells transfected with miR-129-5p inhibitor plasmid), the si-ZIC2 group (DU145 cells transfected with si-ZIC2 plasmid), and the miR-129-5p inhibitor + si-ZIC2 group (DU145 cells transfected with miR-129-5p inhibitor and si-ZIC2 plasmid). The transfection sequences were constructed by Shanghai Sangon Biotech Company (Shanghai, China). Before transfection, cells were cultured in 6-well plates for 24 h. When cell density reached 30–50%, the cells were transfected following the manufacturer’s instructions of lipofectamin 2000 (11668-019, Invitrogen, New York, CA, USA). A total of 250 μL serum-free Opti-minimal essential medium (MEM; 51985042, Gibco, Gaithersburg, MD, USA) was used for dilution of 100 pmol plasmid (the final concentration was 50 nM), mixed gently and incubated for 5 min. Another 250 μL serum-free medium Opti-MEM was used to dilute 5 μL lipofectamin 2000, mixed gently and incubated for 5 min. These two mixtures were incubated for 20 min, added to the cell culture wells, and cultured within 5% CO_2_ at 37 °C for 6–8 h. Next, a complete medium was used for incubation for 24–48 h for following experiments.

### Reverse transcription quantitative polymerase chain reaction (RT-qPCR)

The total RNA was extracted from the transfected cells in strict accordance with the instructions of the TRIZOL kit (15596-018, Beijing Solarbio Science & technology Co., Ltd., Beijing, China), and RNA concentration was determined. The primers used in this study were synthesized by Dalian TaKaRa Corporation (Dalian, Liaoning, China) (Table 1). The reverse transcription was conducted following the instructions of cDNA Reverse Transcription Kit (K1622, Beijing Reanta Biotechnology Co., Ltd., Beijing, China). RT-qPCR was performed using a fluorescence quantitative PCR instrument (ViiA 7, Sun Yat-sen University DAAN GENE Co., Ltd., Guangzhou, Guangdong, China). U6 was used as the internal reference gene and the relative expression of miR-129-5p was calculated. With glyceraldehyde-3-phosphate dehydrogenase (GAPDH) used as the primer of internal reference, the relative expression of target genes (ZIC2, Wnt, β-catenin, E-cadherin and vimentin) was calculated by relative quantitative method (2^−ΔΔCt^) [25].

**Table 1:** Primer sequences for RT-qPCR

Gene	Sequence (5′–3′)
miR-129-5p	F: CAAAAAGCGGACAGG
	R: CAGTGCGTGTCGTGGAGT
ZIC2	F: GAGGGCACCTTGTGATCATGT
	R: ACAGGGTGGGAAAGAACGTG
Wnt	F: CAGAAGGACCTTGTTTGCCAGG
	R: CCTCAGGGTATTGCTGGACAAC
β-Catenin	F: CAAGACCTCGTGCTCCAGTTAG
	R: GACCAAAAGGTGATGCTGGACAG
E-Cadherin	F: TGCCCAGAAAATGAAAAAGG
	R: GTGTATGTGGCAATGCGTTC
Vimentin	F: GAGAACTTTGCCGTTGAAGC
	R: GCTTCCTGTAGGTGGCAATC
U6	F: CTCGCTTCGGCAGCACA
	R: AACGCTTCACGAATTTGCGT
GAPDH	F: ACCCAGAAGACTGTGGATGG
	R: TCTAGACGGCAGGTCAGGTC

*RT-qPCR* reverse transcription quantitative polymerase chain reaction, *miR-129-5p* micro RNA-129-5p, *ZIC2* zinc-finger protein of the cerebellum 2, *GAPDH* glyceraldehyde-3-phosphate dehydrogenase, *F* forward, *R* reverse

### Western blot analysis

After 48 h of culture, cells of each group were lysed with a protein lysis buffer for 30 min at 4 °C and shaken once every 10 min. After centrifugation at 25,764×*g* for 20 min at 4 °C, the supernatant was collected and used as the protein extraction solution. The protein concentration was determined using a bicinchoninic acid (BCA) kit (20201ES76, YEASEN Biotech Co., Ltd., Shanghai, China). The protein was separated by polyacrylamide gel electrophoresis (PAGE), transferred onto a polyvinylidene fluoride (PVDF) membrane by wet transfer method, and blocked with 5% bovine serum albumin (BSA) for 1 h. The membrane was probed with the primary antibodies; rabbit anti-human antibodies to ZIC2 (1:2000, ab150404), Wnt3a (1:1000, ab28472), β-catenin (1:4000, ab6302), p-β-catenin (1:500, ab75777), E-cadherin (1:20,000, ab40772), N-cadherin (1:1000, ab76057), vimentin (1:2000, ab92547), VEGF (1:2000, ab32152), CD31 (1:5000, ab76533), and GAPDH (1:500, ab9485) overnight at 4 °C. After being washed three times with tris-buffered saline tween (TBST) (each time for 5 min), the membrane was probed with HRP-labeled goat anti-rabbit IgG (1:10,000, ab6721) for 1 h at room temperature. All antibodies were bought from Abcam (Cambridge, UK). Subsequently, the membrane was washed three times with TBST (each time for 5 min) and developed. The ImageJ 1.48u software (National Institutes of Health, Bethesda, MD, USA) was used for protein quantitative analysis by computing the ratio of gray value of each protein to that of the internal reference. Each experiment was repeated three times independently.

### 3-(4,5-Dimethylthiazol-2-yl)-2,5-diphenyltetrazolium bromide (MTT) assay

After transfection for 48 h, the cells were counted and seeded in 96-well plates at a ratio of 3 × 10^3^–6 × 10^3^ cells/well (100 μL/well). Six replicate wells were prepared. At the 24th h, 48th h, and 72nd h, cells were incubated with 20 μL prepared 5 mg/mL MTT solution at 37 °C for 2 h. Next, 15 μL Dimethyl Sulphoxide (DMSO; WBBB011a, Beijing Qiangxin Biorepublic Co., Ltd., Beijing, China) was then added to each well. The optical density (OD) value was obtained at 570 nm using a microplate reader (NYW-96M, Beijing Nuoyawei Instrument Co., Ltd., Beijing, China). Each experiment was conducted for three times. A cell viability curve was plotted using the time points at 24th h, 48th h, and 72nd h as abscissa and OD value as ordinate. The cell viability was calculated as follows = OD value of treated cells/OD value of control cells × 100% [26].

### Transwell assay

Cells were starved in serum-free medium for 24 h and detached. Next the cells were resuspended in serum-free Opti-MEMI (31985008, Nanjing SenBeiJia Biological Technology Co., Ltd., Nanjing, Jiangsu, China) containing 10 g/L BSA, and the cell density was adjusted into 3 × 10^4^ cells/mL. A transwell chamber was placed in a 24-well plate, and the bottom membrane on the apical chamber was coated with diluted Matrigel (40111ES08, YEASEN Biotech Co., Ltd., Shanghai, China) at a ratio of 1:8, and then air-dried. Totally, 200 μL of cell suspension was added into the apical chamber coated with Matrigel, and 600 μL of Roswell Park Memorial Institute (RPMI) 1640 culture medium with 20% FBS was added to the basolateral chamber. After 24 h of routine culture, the cells on the apical chamber were removed using cotton swabs, fixed using 4% paraformaldehyde for 15 min, and stained with methanol-prepared 0.5% crystal violet solution for 15 min. Five visual fields were randomly selected and photographed (200×) under an inverted microscope (XDS-800D, Shanghai CIKONG Optical Instrument Co., Ltd., Shanghai, China). The number of cells that had penetrated the membrane was counted and the number of cells in each field was calculated to determine cell migration and invasion. Three replicates were set for all groups. This experiment was repeated for three times to obtain an average value.

### Matrigel angiogenesis assay

Matrigel (356234, Shanghai ShanRan Biotech Co., Ltd., Shanghai, China) was placed in a freezer at 4 °C overnight to melt into a yellow gel. A total of 70 μL (0.5 mmol/L thickness) of the yellow gel was rapidly added to a pre-cooled 96-well plate with a pre-chilled micropipette. Next, the plate was incubated for about 30 min at 37 °C until the Matrigel was frozen. After 48 h of infection, the cells were dissociated into a cell suspension. The cell suspension at 1 × 10^5^ cells/mL was seeded into the wells coated with Matrigel and cell culture medium for the corresponding group was added to each well. The plates were incubated for 18 h, and then photographed under a low power microscopy system. Three fields were randomly selected from each well and the number of blood vessels formed in each field was calculated. Each experiment was carried out three times.

### Flow cytometry

After transfection, the DU145 cells were treated with ethylene diamine tetraacetic acid (EDTA)-free trypsin, collected in a flow tube and centrifuged. The cells were centrifuged again, and then the supernatant was removed. According to the instructions of Annexin-V-fluorescein isothiocyanate (FITC) Cell Apoptosis Detection Kit (40302ES60, YEASEN Biotech Co., Ltd., Shanghai, China), the Annexin-V-FITC/propodium iodide (PI) staining solution was prepared by dilution of the Annexin-V-FITC, PI, and 4-(2-hydroxyethyl)-1-piperazineethanesulfonic acid (HEPES) buffer solution at a ratio of 1:2:50. Every 100 μL staining solution was used to re-suspend 1 × 10^6^ cells, and the cells were shaken fully, incubated for 15 min, added with 1 mL HEPES buffer and mixed well. Cell apoptosis was determined by using 525 nm and 620 nm band pass filters to detect the fluorescence of FITC and PI at an excitation wavelength of 488 nm.

### In vivo xenograft assay

Male BALB/c nude mice (aged 4–6 weeks and weighing 16–22 g) were used. All mice were housed in a humidity-controlled (50–60%) room on a 12/12 h light/dark cycle with ad libitum access to chow and drinking water. After 24 h of transfection, stably transfected DU-145 cells were detached with trypsin, resuspended in serum-free 1640 medium and counted. A total of 1.5 × 10^6^ DU-145 cells were implanted subcutaneously on the back of the nude mice (200 μL suspension). The growth of the resultant tumor was observed every 7 days starting from the 7th day. The volume (V) of the tumor was calculated using the formula V (mm^3^) = (D^2^ × L)/2, where L is the length, and D is the width of the tumor. The development of solid tumors was monitored for up to 35 days post xenotransplantation. All mice were euthanized, and the tumor was excised and weighed.

### Statistical analysis

SPSS21.0 statistical software (SPSS, IBM, Armonk, NY, USA) was used to analyze the statistical data. The enumeration data were expressed as the number of cases or percentages. The comparisons were carried out using a Chi square test or Fisher’s exact test. The measurement data were summarized as mean ± standard deviation and. Data between 2 groups were analyzed using a *t* test, while those among multiple groups were compared using one-way analysis of variance (ANOVA). Pairwise comparisons of mean values were made using Tukey test. α = 0.05 was taken as the test level, and *p* < 0.05 was considered as statistically significant.

## Results

### miR-129-5p affects PCa by regulating ZIC2 and the Wnt/β-catenin signaling pathway

**Fig. 1:**
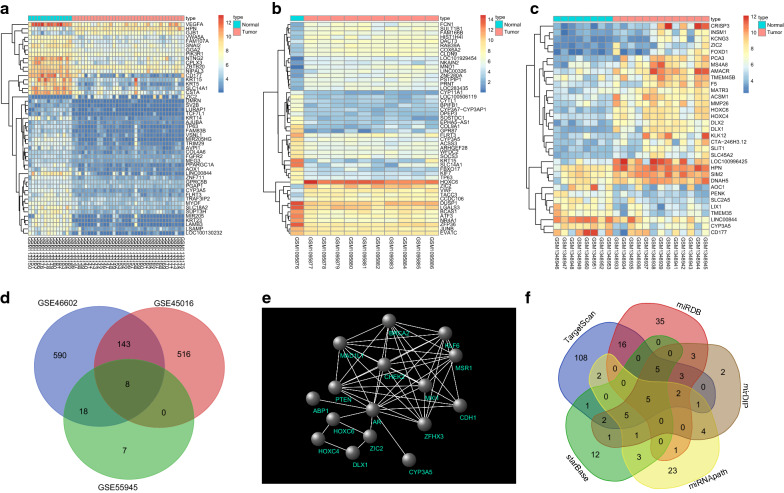
miR-129-5p influences PCa by regulating ZIC2 via the Wnt/β-catenin signaling pathway. **a**–**c** The heat maps depicting DEGs in PCa-related microarray datasets, with the X label indicating the sample number, the Y label indicating the gene name; the left-side dendrogram indicating gene expression clustering, with each small square in the figure representing the expression of one gene in one sample, and the histogram in the upper right representing the color gradation; **d** venn diagram analysis for DEGs in PCa-related microarray datasets, the 3 circles with different colors in the figure respectively represent the number of DEGs in the 3 PCa microarray datasets, the overlapping area indicates the intersection among the three microarray datasets; **e** gene interaction network map, each small ball in the figure denotes one gene, and the line between two small balls indicates an interaction; **f** predicted miRNAs that regulate ZIC2, and results from five databases are included. Five colors represent the prediction outcomes of five databases, the overlapping area indicates the intersecting results. *PCa* prostate cancer, *ZIC2* zinc-finger protein of the cerebellum 2, *DEGs* differentially expressed genes

The GEO database was used to identify 3 PCa-related microarray datasets (GSE45016, GSE55945 and GSE46602). Differential analysis of gene expression in PCa samples and normal control samples on these three microarray datasets identified 667 DEGs in GSE45016, 33 DEGs in GSE55945, and 759 DEGs in GSE46602. Figure 1a, b present the heat maps of 50 most significant DEGs in GSE46602 and GSE45016, respectively, and Fig. 1c is the heat map of all DEGs in GSE55945. A Venn diagram analysis was performed for the DEGs obtained from the three datasets to identify the overlapping DEGs (Fig. 1d). It was found that 8 genes were overlapping. Based on MalaCards database, 10 PCa-related known genes with highest scores were obtained (Table 2). In order to further identify the PCa-related genes among the DEGs, 8 obtained DEGs and 10 known PCa-related genes were used for correlation interaction analysis, and a gene interaction network map was plotted (Fig. 1e). The results revealed that the 10 known PCa-related genes were closely clustered and there was a replicating interaction between them. Among the 8 DEGs, ZIC2 and HOXC6 genes not only interacted directly with AR in the known genes, but also interacted with other DEGs. After further retrieving the literatures concerning ZIC2 and HOXC6 genes, it was found that the effect of HOXC6 on PCa had been reported in numerous studies [27–29]. However, the role of ZIC2 in PCa remained unclear. By retrieval of PCa-related signaling pathways, the Wnt/β-catenin signaling pathway was found to have a role in development of PCa [30, 31]. In order to understand the upstream regulatory mechanism of ZIC2 and predict the miRNAs that regulated ZIC2, five databases including TargetScan and miRDB were applied to obtain overlapping results (Fig. 1f). Finally, there were 5 miRNAs in the intersection of the five database predictions. After retrieval of the binding of these 5 miRNAs with ZIC2 in the microRNA.org database, miR-181a, miR-181b, and miR-181c were all found to bind to ZIC2 at the same location, and they were found to have multiple binding sites on ZIC2. The mirSVR score of miR-96-5p and ZIC2 was − 0.3957, whereas miR-129 only bound to ZIC2 at 180 locations with mirSVR score of − 0.7863. Hence, miR-129 was selected for following study. Based on the above analysis and related reports, it was evident that miR-129-5p was likely to affect the development of PCa by targeting ZIC2 and regulating the Wnt/β-catenin signaling pathway.

**Table 2:** PCa-related genes

Symbol	Description	Score	Pubmed IDs
PTEN	Phosphatase and tensin homolog	1445.59	19081794, 20103652, 16778075
CHEK2	Checkpoint kinase 2	1441.94	16551709, 17085682, 15492928
MXI1	MAX interactor 1, dimerization protein	1394.38	7773287, 19042984, 24071797
AR	Androgen receptor	1166.48	7723794, 19042984, 24071797
BRCA2	BRCA2, DNA repair associated	1100.63	11170288, 19042984, 24071797
MSR1	Macrophage scavenger receptor 1	1083.9	12244320, 19042984, 24071797
ZFHX3	Zinc finger homeobox 3	1067.26	16637072, 15750593, 16932943
KLF6	Kruppel like factor 6	1031.73	12651626, 18755691, 15247715
MAD1L1	Mitotic arrest deficient 1 like 1	1008.24	25831061, 11423979, 25781993
CDH1	Cadherin 1	686.47	7585573, 16483154, 17406365

*Symbol* gene abbreviations, *Description* the gene description or gene full name, *Score* this score originates from Solr based GeneCards search engine score, obtained by querying the disease in GeneCards, *Pubmed IDs* PMID number of the related references, *PCa* prostate cancer

### PCa tissues show increased ZIC2 expression and activated Wnt/β-catenin signaling pathway while decreasing miR-129-5p expression

**Fig. 2:**
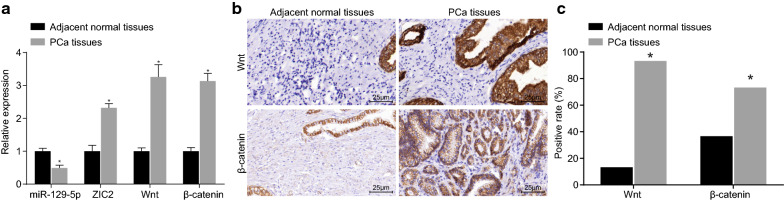
miR-129-5p is poorly-expressed while ZIC2, Wnt3a and β-catenin are highly-expressed in PCa tissues. **a** The miR-129-5p expression and mRNA expression of ZIC2, Wnt3a and β-catenin in PCa tissues and adjacent normal tissues determined by RT-qPCR. Data are summarized as mean ± standard deviation and compared with paired *t* test (n = 60). **b** Immunohistochemical staining of Wnt3a and β-catenin in PCa tissues and adjacent normal tissues, in which brown or yellowish staining represents positive cells. **c** The statistical analysis of **b**. *p < 0.05 vs. the adjacent normal tissues. Positive rates are count data and were compared with the Chi square test

RT-qPCR was used quantify the expression of miR-129-5p, ZIC2, Wnt, and β-catenin in adjacent normal tissues and PCa tissues, and the results (Fig. 2a) showed that compared with the control group, the expression of miR-129-5p in the PCa group was markedly decreased, but the mRNA expression of ZIC2, Wnt, and β-catenin was significantly increased (all *p* < 0.05). These results suggested that PCa tissues showed poorly expressed miR-129-5p while highly expressed ZIC2, Wnt, and β-catenin.

Immunohistochemistry was used to analyze the expression of Wnt3a and β-catenin in PCa tissues (Fig. 2b, c), which revealed that the positive expression rates of Wnt3a and β-catenin in adjacent normal tissues were 13.33% and 36.67%, respectively, whereas these rates in the PCa tissues were 73.33% and 93.33%, respectively. The number of positive cells was obviously increased. All these results indicated that the positive expression rates of Wnt3a and β-catenin in the PCa tissues were significantly increased (all *p* < 0.05).

### miR-129-5p targets ZIC2

**Fig. 3:**
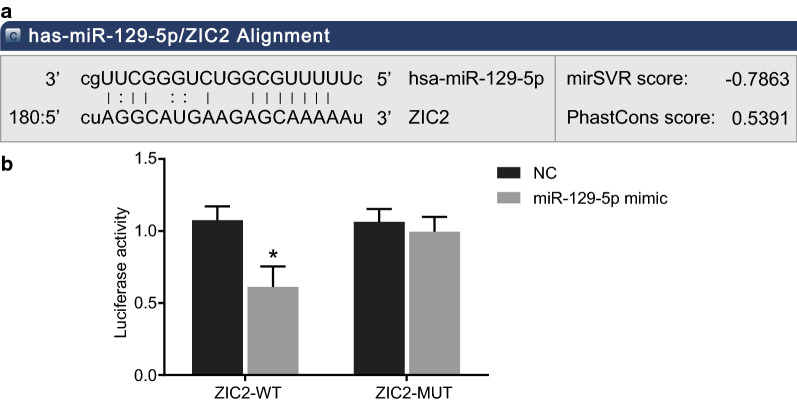
miR-129-5p targets ZIC2. **a** The targeting relationship between ZIC2 and miR-129-5p; **b** the luciferase activity of ZIC2-WT and ZIC2-MUT in each group. *p < 0.05 vs. the NC group. The data are summarized as mean ± standard deviation and analyzed by the *t* test. This experiment was performed three times. *WT* wild-type, *MUT* mutant

Based on bioinformatic analysis, there was a specific binding between the ZIC2 gene sequence and the miR-129-5p sequence, and ZIC2 was a target gene of miR-129-5p (Fig. 3a). The dual-luciferase reporter gene assay was used to verify that ZIC2 was a target of miR-129-5p (Fig. 3b). The experimental results showed that the luciferase signal of ZIC2-Wt in the miR-129-5p mimic group was decreased (*p* < 0.05) as compared with the NC group, with no significant difference in the ZIC2-mut (*p* > 0.05). Therefore, miR-129-5p was demonstrated to specifically bind to ZIC2.

### Overexpression of miR-129-5p dampens the Wnt/β-catenin signaling pathway and EMT process by targeting ZIC2

**Fig. 4:**
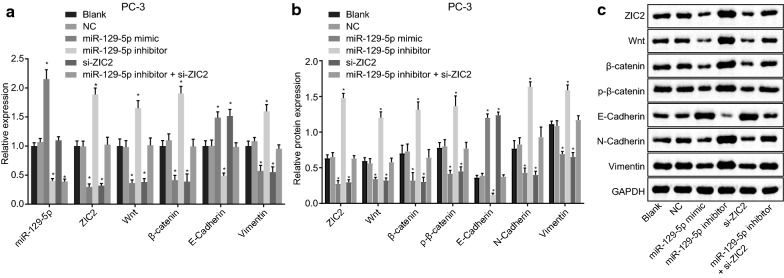
Overexpression of miR-129-5p suppresses the activation of ZIC2-dependent Wnt/β-catenin signaling pathway and EMT in PCa. DU-145 cells were treated with miR-129-5p mimic, miR-129-5p inhibitor or/and si-ZIC2. **a** The miR-129-5p expression and the mRNA expression of ZIC2, Wnt3a, β-catenin, E-cadherin and vimentin in DU-145 cells determined by RT-qPCR. **b**, **c** The protein expression of ZIC2, Wnt3a, β-catenin, E-cadherin and vimentin, and extent of β-catenin phosphorylation in DU-145 cells determined by western blot analysis; *p < 0.05 vs. the blank and NC groups; the experimental data are summarized as mean ± standard deviation; the one-way analysis of variance (ANOVA) was employed to analyze data among groups. This experiment was repeated three times

miR-129-5p expression, mRNA and protein expression of ZIC2, Wnt, β-catenin, E-cadherin and vimentin as well as the extent of β-catenin phosphorylation in cells were evaluated by RT-qPCR (Fig. 4a) and Western blot analysis (Fig. 4b, c). And the results showed that the expression of miR-129-5p in the miR-129-5p mimic group increased (*p* < 0.05), with no significant difference of expression of miR-129-5p in the si-ZIC2 group (*p* > 0.05) when compared with the blank and NC groups. The expression of ZIC2, Wnt, β-catenin and vimentin, as well as the extent of β-catenin phosphorylation were significantly decreased, while that of E-cadherin was significantly increased in the miR-129-5p mimic and si-ZIC2 groups (*p* < 0.05). Compared with the blank and NC groups, the miR-129-5p expression and expression of E-cadherin in the miR-129-5p inhibitor group were significantly lower, while the expression of ZIC2, Wnt, β-catenin and vimentin as well as the extent of β-catenin phosphorylation were significantly enhanced (*p* < 0.05). Relative to the blank and NC groups, the expression of miR-129-5p in the miR-129-5p inhibitor + si-ZIC2 group was markedly decreased (*p* < 0.05), with no significant difference in the expression of ZIC2, Wnt, β-catenin, E-cadherin, and vimentin as well as the extent of β-catenin phosphorylation (*p* > 0.05). Taken together, these results indicated that overexpressed miR-129-5p might hinder the activation of the Wnt/β-catenin signaling pathway by targeting ZIC2.

### Inhibited proliferation of PCa cells is observed after overexpression of miR-129-5p or down-regulation of ZIC2 treatment

**Fig. 5:**
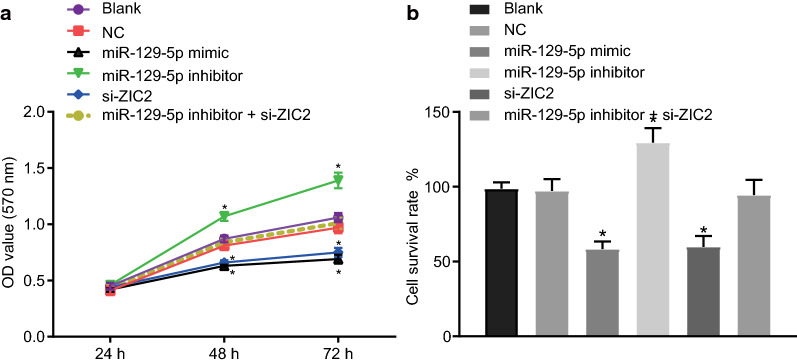
Overexpression of miR-129-5p or down-regulation of ZIC2 inhibits PCa cell proliferation. DU-145 cells were treated with miR-129-5p mimic, miR-129-5p inhibitor or/and si-ZIC2. **a** The OD values at the 24th, 48th, and 72nd h in DU-145 cells. **b** Cell survival rate after 48 h of transfection. The data are summarized as mean ± standard deviation; the repeated measurement analysis of variance was conducted to compare cell proliferation at different time points. The experiment was performed three times. *p < 0.05 vs. the blank and NC groups

MTT assay was applied to investigate effects of miR-129-5 on the proliferation of PCa cells (Fig. 5a). There was no difference in OD value obtained at 24 h between groups (*p* > 0.05). However, the OD values of the miR-129-5p mimic and si-ZIC2 groups at 48 h and 72 h were significantly decreased (*p* < 0.05), while those of the miR-129-5p inhibitor group were significantly increased (*p* < 0.05) as compared with the blank and NC groups. No difference in the OD values of the miR-129-5 inhibitor + si-ZIC2 group at 48 h and 72 h after transfection was observed (*p* > 0.05). Cell survival rate in all groups showed consistent trends (Fig. 5b). These results suggested the proliferation of PCa cells might be inhibited by over-expressed miR-129-5p or down-regulated ZIC2.

### Cell migration and invasion PCa cells are repressed after overexpression of miR-129-5p or down-regulation of ZIC2 treatment

**Fig. 6:**
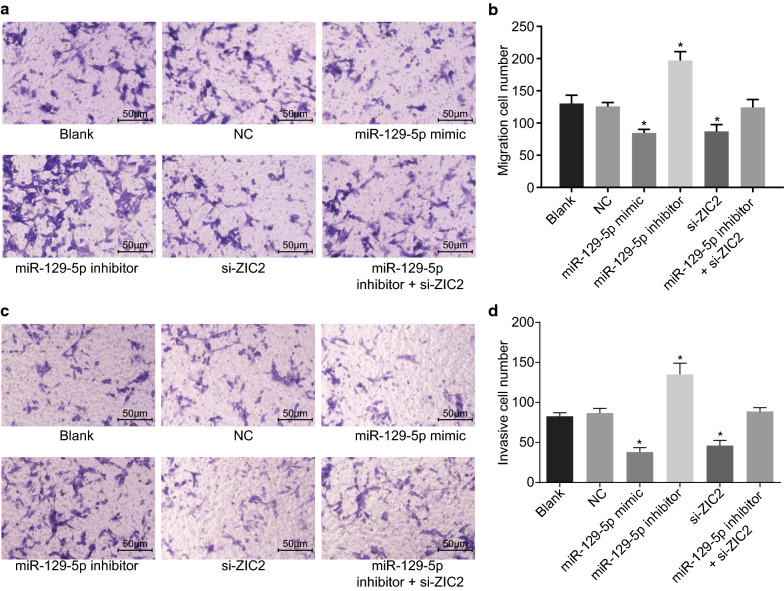
Overexpressed miR-129-5p or silencing ZIC2 inhibits PCa cell migration and invasion. DU-145 cells were treated with miR-129-5p mimic, miR-129-5p inhibitor or/and si-ZIC2. **a** Cell migration within each group (×200). **b** The migration cell number. **c** The cell invasion (×200). **d** The invasive cell number in each group. *p < 0.05 vs. the blank and NC groups. The data are summarized as mean ± standard deviation and compared using one-way analysis of variance. The experiment was repeated three times and the average value was obtained

Transwell assay was used to evaluate the effect of miR-129-5p on migration (Fig. 6a, b) and invasion (Fig. 6c, d) of PCa cells. Compared with the blank and NC groups, cell migration and invasion in the miR-129-5p mimic and si-ZIC2 groups were significantly decreased (all *p* < 0.05) while those in the miR-129-5p inhibitor group were significantly elevated (all *p* < 0.05). No significant difference in cell migration and invasion in the miR-129-5p inhibitor + si-ZIC2 group was observed (*p* > 0.05). The results indicated that cell migration and invasion of PCa was attenuated by over-expression of miR-129-5p or down-regulation of ZIC2.

### Repressed angiogenesis of PCa cells is attributed to overexpressed miR-129-5p or down-regulated ZIC2

**Fig. 7:**
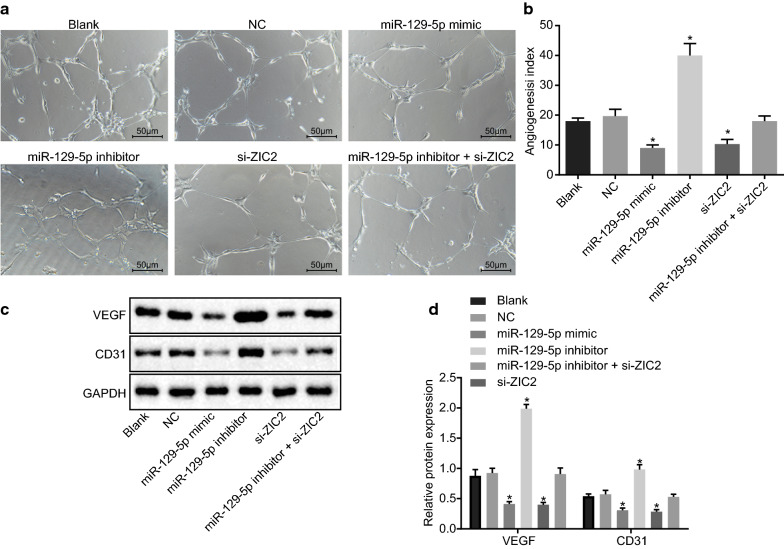
Angiogenesis of PCa cells is blocked by overexpressed miR-129-5p or down-regulated ZIC2. DU-145 cells were treated with miR-129-5p mimic, miR-129-5p inhibitor or/and si-ZIC2. **a** The angiogenesis in each group; **b** the angiogenesis indexes in each group; **c** the protein expression of VEGF and CD31 in each group; **d** the statistical analysis of **c**. *p < 0.05 vs. the blank and NC groups. The data are summarized as mean ± standard deviation. The experiment was repeated three times and the data were compared using the one-way analysis of variance. *VEGF* vascular endothelial growth factor, *GAPDH* glyceraldehyde-3-phosphate dehydrogenase

Matrigel assay was used to analyze the impact of miR-129-5p expression on angiogenesis of PCa cells, and the results (Fig. 7a, b) showed that, the miR-129-5p mimic and si-ZIC2 groups showed significantly decreased angiogenesis (*p* < 0.05), while miR-129-5p inhibitor group showed significantly enhanced angiogenesis (*p* < 0.05) when compared to the blank and NC groups. No significant difference in angiogenesis was found in the miR-129-5p inhibitor + si-ZIC2 group (*p* > 0.05).

Western blot analysis was used to analyze the expression of VEGF and CD31 in PCa cells (Fig. 7c, d). The protein expression of VEGF and CD31 in the miR-129-5p mimic and si-ZIC2 groups was found to be significantly decreased (*p* < 0.05), while that in the miR-129-5p inhibitor group was significantly higher (*p* < 0.05) as compared with the blank and NC groups. No significant difference, however, was found in the protein expression of VEGF and CD31 in the miR-129-5p inhibitor + si-ZIC2 group (*p* > 0.05). In summary, these findings indicated that overexpressed miR-129-5p or down-regulated ZIC2 suppressed angiogenesis in PCa cells.

### Overexpressed miR-129-5p or down-regulated ZIC2 leads to PCa cell apoptosis

**Fig. 8:**
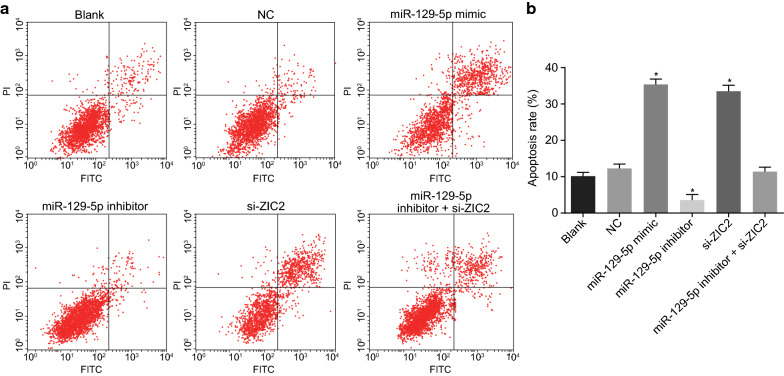
Overexpressed miR-129-5p or down-regulated ZIC2 plays a positive role in apoptosis of PCa cells. DU-145 cells were treated with miR-129-5p mimic, miR-129-5p inhibitor or/and si-ZIC2. **a** The apoptosis in each group; **b** the apoptosis rate in each group. *p < 0.05 vs. the blank and NC groups. The data are summarized as mean ± standard deviation and the one-way analysis of variance was used for comparison. This experiment was performed three times

Flow cytometry was used to examine the effect of miR-129-5p on apoptosis of PCa cells (Fig. 8a, b). The apoptosis rate of the miR-129-5p mimic and si-ZIC2 groups was significantly potentiated (*p* < 0.05), while that of the miR-129-5p inhibitor group was significantly diminished (*p* < 0.05), as compared with the blank and NC groups. No statistically significant difference was evident in the apoptosis rate of the miR-129-5p inhibitor + si-ZIC2 group (*p* > 0.05). These results showed overexpressed miR-129-5p or down-regulated ZIC2 might promote the apoptosis of PCa cells.

### miR-129-5p overexpression or ZIC2 silencing promotes cell tumorigenesis in PCa

**Fig. 9:**
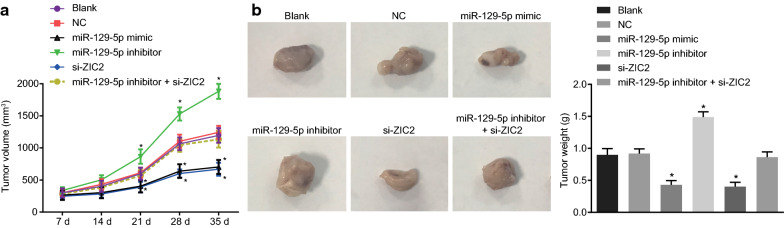
miR-129-5p overexpression or ZIC2 silencing promotes tumorigenesis. Nude mice were treated with miR-129-5p mimic, miR-129-5p inhibitor or/and si-ZIC2. **a** The tumor growth curve of nude mice in each group. **b** Representative images and tumor weights of nude mice. *p < 0.05 vs. the blank and NC groups. The data are summarized as mean ± standard deviation and the one-way analysis of variance was used for comparison. n = 10

Finally, we examined the function of miR-129-5p overexpression or ZIC2 silencing on tumorigenesis. As shown in Fig. 9, both the tumor volume and weight were significantly lower in the miR-129-5p mimic and si-ZIC2 groups than that in the blank and NC groups (*p* < 0.05). Conversely, the tumor volume and weight were significantly higher in the miR-129-5p inhibitor group than that in the blank and NC groups (*p* < 0.05). These data illustrated that ZIC2 served as an oncogene in PCa and miR-129-5p played a tumor-suppressive role.

## Discussion

PCa is one of the most prevalent carcinomas among men, resulting in a high number of cancer-related deaths [32]. miRNAs have been implicated in biological processes such as cell proliferation, differentiation, development, apoptosis, and metabolism, and their alterations have been found in various cancers and participate in pathogenesis of cancers [33, 34]. Importantly, miR-129-5p has been demonstrated to express aberrantly in primary tumors of human PCa and prostate control specimens [35]. Therefore, this study investigated the function of miR-129-5p in PCa, and it was found that up-regulated miR-129-5p could inhibit EMT and angiogenesis in PCa.

Initially, the results obtained from present study revealed that up-regulation of miR-129-5p could attenuate EMT and angiogenesis of PCa. A previous study reported that miR-129-5p modulated EMT in tubular epithelial cells by targeting the gene PDPK1 [36]. β-Catenin, p-β-catenin, N-cadherin and vimentin are well established as indicators of EMT, and accordingly, the poor expression of β-catenin in chondrosarcoma cells of the miR-129-5p group has been observed, which further suppressed the cell proliferation, migration and promoted apoptosis [21]. Similarly, over-expression of miR-129-5p in the breast cancer cell line MCF-7 was found to significantly induce E-cadherin and suppress N-cadherin and vimentin expression [37]. Corroborating these findings, we noted that poor expression of β-catenin, N-cadherin, and vimentin and high expression of E-cadherin were markers of PCa inhibition. Additionally, up-regulation miR-195 has been found to reduce VEGF level by blocking VEGF receptor 2 signaling in endothelial cells and consequently inhibiting angiogenesis, consistent with our findings [38].

Additionally, miR-129 might play a role in inhibition of viability, proliferation, migration and invasion of PCa cells via directly suppressing E26 transformation specific-1 (ETS1), which was also provided new train of thought for us to popularize the carcinogenesis of PCa [39]. In laryngeal cancer, miR-129-5p can mediate cell proliferation, invasiveness, and migration by suppressing the expression of STAT3 [40]. Another study suggested that miR-129-5p mediates FNDC3B to suppress proliferation, migration and invasion of glioblastoma cells U87 cells [41]. Overexpression of miR-129-5p alone has been found sufficient to promote apoptosis [42]. Consistent with these findings, the current study demonstrated that up-regulated miR-129-5p could inhibit proliferation, migration, and invasion while promoting apoptosis of PCa cells.

Here, we showed that an operant mechanism of miR-129-5p in PCa involves impairment of the Wnt/β-catenin signaling pathway via down-regulation of ZIC2. The Wnt/β-catenin signaling pathway, central to tissue development in embryos and tissue maintenance in adults, is a major up-regulated signaling pathway in castration-resistant PCa [43]. Vimentin and E-cadherin are established as Wnt/β-catenin signaling pathway-related factors [44]. The Wnt/β-catenin signaling pathway is blocked upon down-regulation of the EMT marker vimentin [45]. The up-regulated E-cadherin expression in microwells was found following with a downregulation of the Wnt signaling pathway and the deficiency of nuclear β-catenin as well [46]. Consistent with former results, our data indicated over-expressed miR-129-5p and down-regulated ZIC2 reduced the expression of Wnt, β-catenin and vimentin, but restored the expression of E-cadherin, which further indicated the inhibitory role of over-expressed miR-129-5p or down-regulated ZIC2 on the Wnt/β-catenin signaling pathway.

## Conclusion

**Fig. 10:**
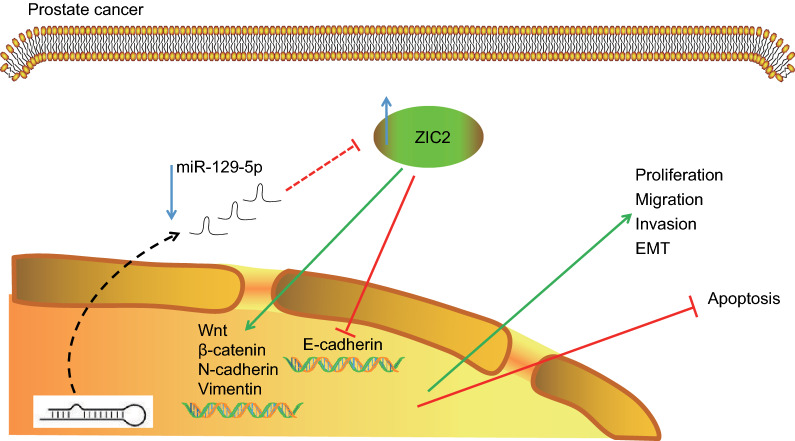
The molecular mechanism of miR-129-5p regulation of ZIC2 expression and the Wnt/β-catenin signaling pathway in PCa. In PCa, the expression of miR-129-5p was significantly decreased, while the expression of ZIC2 was significantly increased. miR-129-5p targeted ZIC2 and inhibited the expression of ZIC2. The up-regulated miR-129-5p could suppresses the expression of ZIC2, leading to reduced expression of Wnt, β-catenin, N-cadherin and vimentin with increase in E-cadherin expression. Eventually, up-regulated miR-129-5p significantly inhibited cell proliferation, migration, invasion and EMT while promoting PCa cell apoptosis

In a conclusion, elevated miR-129-5p was found to block the activation of the Wnt/β-catenin signaling pathway in PCa, consequently inhibiting EMT and angiogenesis via targeting ZIC2 (Fig. 10). miR-129-5p can be considered as a new therapeutic target for PCa therapy. The down-regulation of miR-129-5p can promote ZIC2 expression via activating the Wnt/β-catenin signaling pathway, and further enhanced the expression of Wnt, β-catenin, N-cadherin and vimentin and inhibited the expression of E-cadherin, thus resulting in cancer cell proliferation, invasion, migration along with EMT and angiogenesis and reduced apoptosis. Overexpression of miR-129-5p may reverse these events, thus limiting the growth of PCa. However, further studies with different disease models and larger cohorts are essential to validate these findings and expand the translational potential of this direction.

## Data Availability

The datasets generated/analyzed during the current study are available.

## Abbreviations

PCa: prostate cancer
VEGF: vascular endothelial growth factor
EMT: epithelial–mesenchymal transition
ZIC: zinc-finger protein of the cerebellum
OPA: odd-paired
GS: Gleason score
