# Correction to: Inactivation of the Wnt/β-catenin signaling pathway underlies inhibitory role of microRNA-129-5p in epithelial–mesenchymal transition and angiogenesis of prostate cancer by targeting ZIC2

**DOI:** 10.1186/s12935-021-01945-5

**Published:** 2021-04-26

**Authors:** Zhenming Jiang, Yuxi Zhang, Xi Chen, Pingeng Wu, Dong Chen

**Affiliations:** 1 Department of Urology, The First Hospital of China Medical University, No. 155, Nanjing North Street, Heping District, Shenyang, 110001 Liaoning People’s Republic of China; 2 Department of Urology, People’s Hospital of Datong Hui and Tu Autonomous County, No. 1, Wenhua Road, Qiaotou Town, Datong Hui and Tu Autonomous County, Xining, 810100 Qinghai People’s Republic of China; 3 Department of Pharmacy, The First Hospital of China Medical University, Shenyang, 110001 People’s Republic of China; 4 Central Lab, The First Hospital of China Medical University, Shenyang, 110001 People’s Republic of China

## Correction to: Cancer Cell Int (2019) 19:271 10.1186/s12935-019-0977-9

Following the publication of the original article [1], we were notified of a few minor mistakes in Figures 6 and 7.

The corrected Figs. 6, 7 can be found below:

**Fig. 6:**
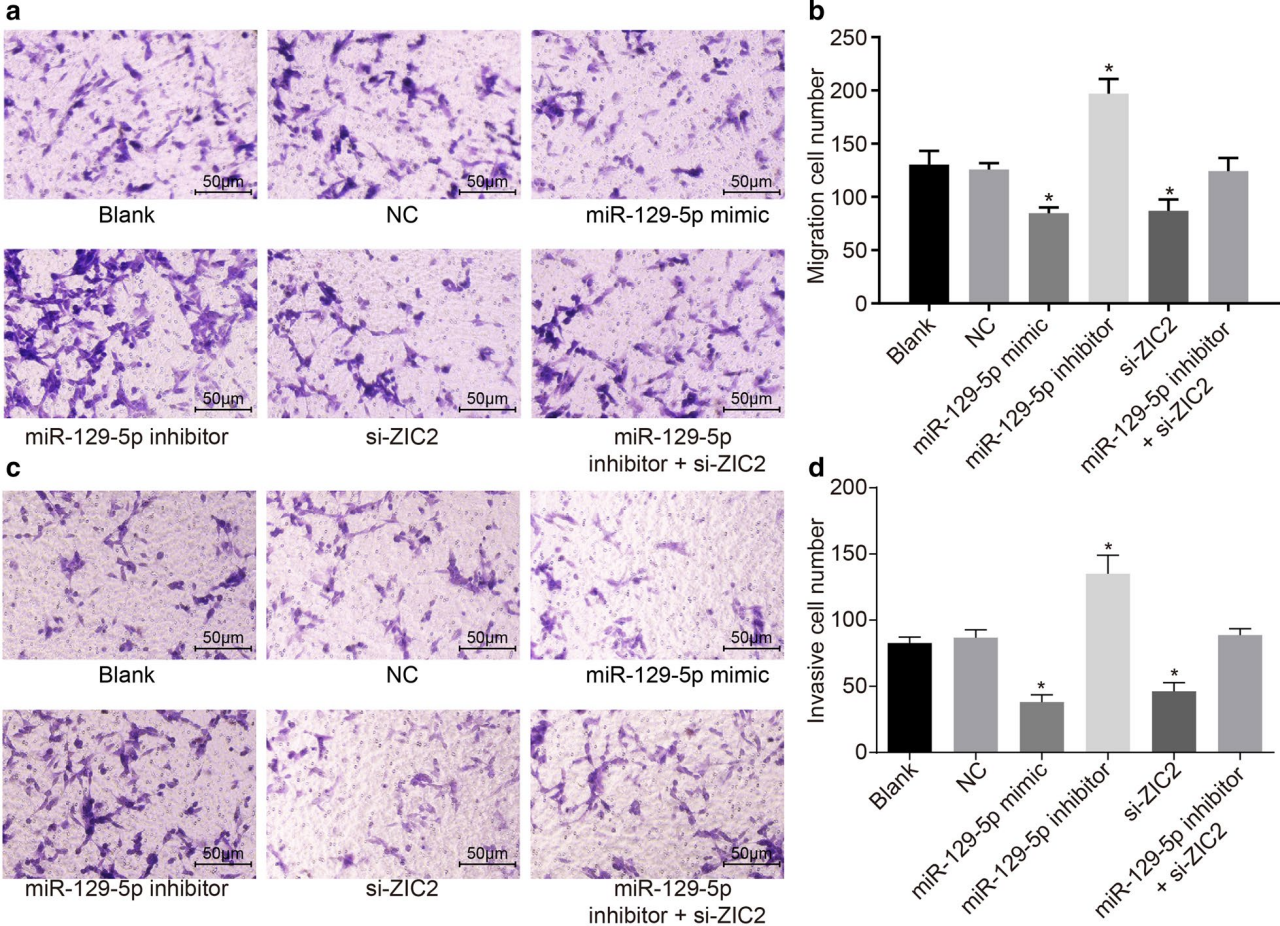
Overexpressed miR-129-5p or silencing ZIC2 inhibits PCa cell migration and invasion. DU-145 cells were treated with miR-129-5p mimic, miR-129-5p inhibitor or/and si-ZIC2. **a** Cell migration within each group (× 200). **b** The migration cell number. **c** The cell invasion (× 200). **d** The invasive cell number in each group. *p < 0.05 vs. the blank and NC groups. The data are summarized as mean ± standard deviation and compared using one-way analysis of variance. The experiment was repeated three times and the average value was obtained

**Fig. 7:**
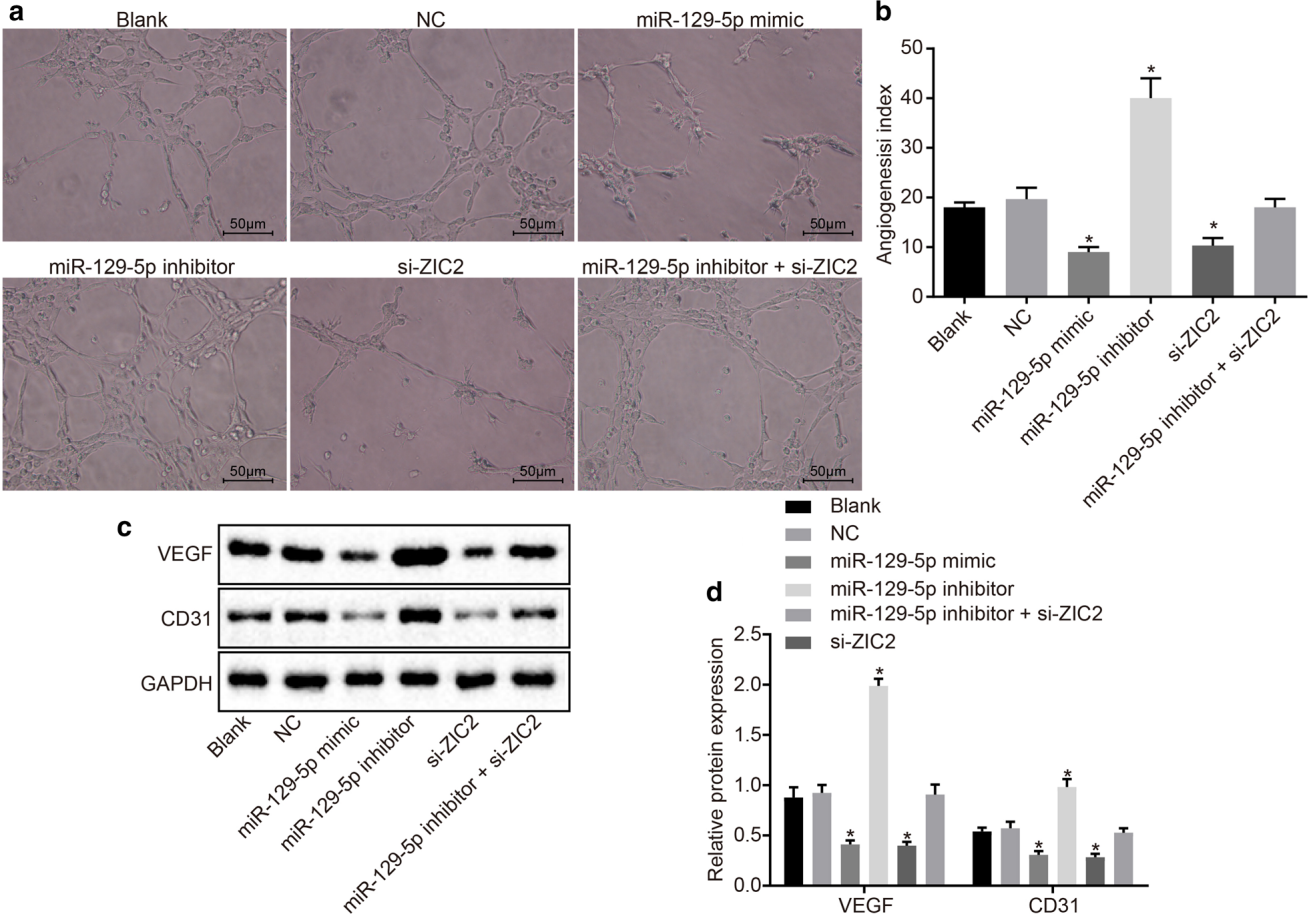
Angiogenesis of PCa cells is blocked by overexpressed miR-129-5p or down-regulated ZIC2. DU-145 cells were treated with miR-129-5p mimic, miR-129-5p inhibitor or/and si-ZIC2. a The angiogenesis in each group; b the angiogenesis indexes in each group; c the protein expression of VEGF and CD31 in each group; d the statistical analysis of c. *p < 0.05 vs. the blank and NC groups. The data are summarized as mean ± standard deviation. The experiment was repeated three times and the data were compared using the one-way analysis of variance. *VEGF* vascular endothelial growth factor, *GAPDH* glyceraldehyde-3-phosphate dehydrogenase
